# Regulation of persistent sodium currents by glycogen synthase kinase 3 encodes daily rhythms of neuronal excitability

**DOI:** 10.1038/ncomms13470

**Published:** 2016-11-14

**Authors:** Jodi R. Paul, Daniel DeWoskin, Laura J. McMeekin, Rita M. Cowell, Daniel B. Forger, Karen L. Gamble

**Affiliations:** 1 Department of Psychiatry, University of Alabama at Birmingham, 1720 7th Avenue South, Birmingham, Alabama 35294, USA; 2 Department of Mathematics, University of Michigan, 530 Church Street, Ann Arbor, Michigan 48109, USA

## Abstract

How neurons encode intracellular biochemical signalling cascades into electrical signals is not fully understood. Neurons in the central circadian clock in mammals provide a model system to investigate electrical encoding of biochemical timing signals. Here, using experimental and modelling approaches, we show how the activation of glycogen synthase kinase 3 (GSK3) contributes to neuronal excitability through regulation of the persistent sodium current (*I*_NaP_). *I*_NaP_ exhibits a day/night difference in peak magnitude and is regulated by GSK3. Using mathematical modelling, we predict and confirm that GSK3 activation of *I*_NaP_ affects the action potential afterhyperpolarization, which increases the spontaneous firing rate without affecting the resting membrane potential. Together, these results demonstrate a crucial link between the molecular circadian clock and electrical activity, providing examples of kinase regulation of electrical activity and the propagation of intracellular signals in neuronal networks.

## Abstract (web summary)

It is not clear how circadian biochemical cascades are encoded into neural electrical signals. Here, using a combination of electrophysiology and modelling approaches in mice, the authors show activation of glycogen synthase kinase 3 modulates neural activity in the suprachiasmatic nuclei via regulation of the persistent sodium current, INaP.

Much information processing in the brain occurs through electrical signalling, where the opening and closing of ion channels controls the properly timed firing of action potentials (APs). At the same time, biochemical networks within neurons separately regulate transcription, translation and post-translational modifications to determine cell fate, time cellular events and control cell size. How neurons integrate information between the two levels has remained a key open problem in neuroscience.

An important model system for studying this phenomenon is the neuronal network within the suprachiasmatic nuclei (SCN) of the hypothalamus, which forms the central circadian (24-h) pacemaker^1^. Within the neurons of the SCN, an intracellular clock generates daily rhythms through regulation of transcription factors, kinases, and other signalling molecules^2^. Timing information is relayed to the rest of the body through a variety of electrical behaviours and signals produced by SCN neurons^3^. Understanding how cellular timekeeping and electrical activity are integrated is an essential ongoing question in the field. Since many of the molecular and electrical components of the clock are well characterized, the SCN is a perfect system for investigating the greater question in neuroscience of how intracellular biochemical information is electrically encoded.

One potential link between cellular biochemistry and electrical activity is glycogen synthase kinase 3 (GSK3). GSK3 activation enhances the spontaneous firing rate (SFR)^4^, is regulated in a circadian manner^5^,^6^, and modulates molecular timekeeping within SCN neurons^5^, providing a potential link between the molecular and electrical activities of the SCN. Here, we test the hypothesis that GSK3 regulates specific ionic currents that affect the spontaneous firing of APs. As GSK3 is implicated in many other intracellular processes (for example, inflammatory pathways, neurotrophic signalling, Wnt and mTOR cascades)^7^,^8^,^9^,^10^, GSK3 could provide a universal link between the biochemical state of the cell and its electrical activity.

It has been previously reported that several specific ionic currents are activated in a circadian manner^3^, and thus, may provide a primary mechanism for controlling circadian variation in electrical activity. The presumed mechanism of circadian regulation of many currents, however, is through transcriptional regulation of their channels, which is energetically inefficient. Another challenge to this hypothesis is that experimental and computational studies implicate sodium currents as the most important regulators of firing rate in SCN neurons^11^,^12^, but no evidence to support a day–night difference in these currents has been presented to date. Here, we propose another mechanism for the regulation of the electrical activity of a neuron by molecular signalling through GSK3. Using a combination of mathematical modelling and experimental work, we show that GSK3 efficiently regulates the firing rate of the cell by modulation of a persistent sodium current without causing the significant depolarization that could signal back to the molecular circadian clock.

## Results

### GSK3 inhibition reduces spiking in a time-specific manner

**Figure 1:**
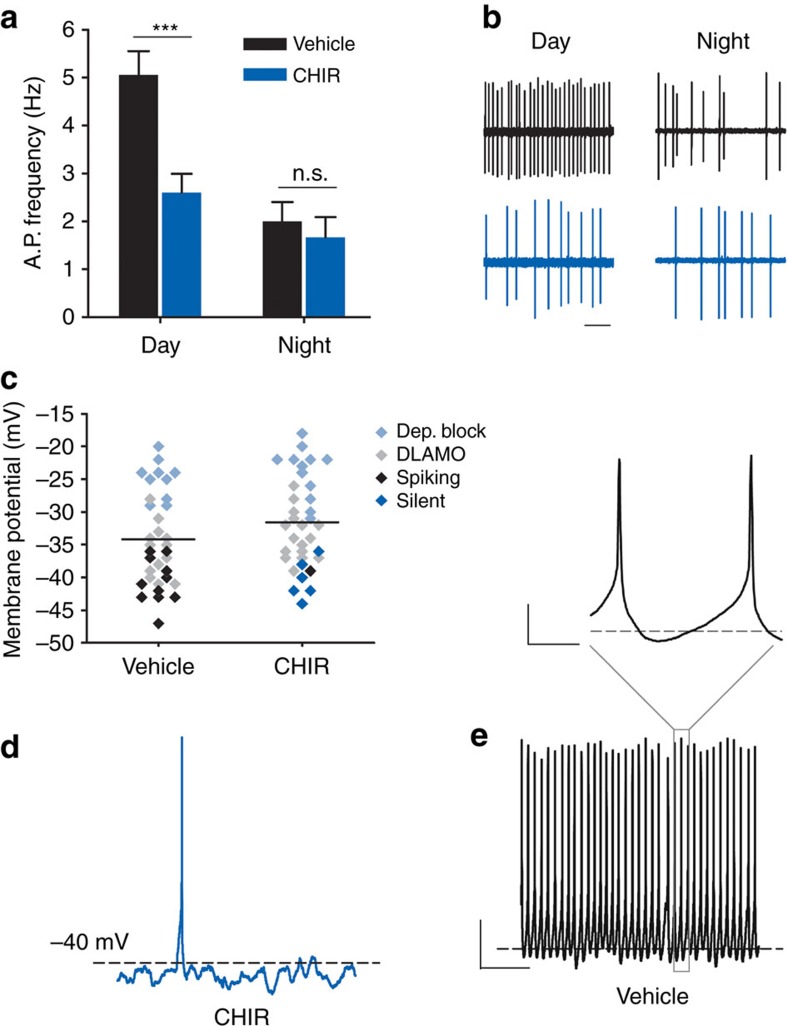
GSK3 inhibition suppresses excitability. (**a**) Spontaneous AP frequencies (mean±s.e.m.) from 2-min loose-patch recordings of neurons treated with vehicle (0.002% DMSO; black) or CHIR-99021 (CHIR, 1 μM; blue) during the day (ZT 4–8) or early-night (ZT 14–18). CHIR significantly suppressed SCN activity during the day (***P<0.001) but not the night (NS; *P*=0.608). Two-way ANOVA; treatment X time interaction, *F*_(1,146)_=5.772, *P*=0.018. From left to right, *n*=38 cells, 4 animals; 41 cells, 4 animals; 38 cells, 3 animals; 33 cells and 3 animals. (**b**) Representative cell-attached loose-patch traces (10 s) from each group. Scale bar, 1 s. (**c**) Dot plot of RMP for individual SCN neurons treated with vehicle or CHIR during the mid-day (ZT 4–8). Symbol colour indicates subgroup phenotype as indicated in the legend. Lines indicate means for Vehicle and CHIR groups. There was no difference in RMP between groups. Independent samples *t*-test; *t*_(67)_=−1.521, *P*=0.133; *n*=34–35 cells, 5–6 animals per group. (**d**,**e**) Representative current clamp traces (5 s) of SCN neurons from each group in **c**. Scale bar, 10 mV, 1 s. Inset shows depolarizing ramp between APs in control cells. Scale bar, 10 mV, 50 ms.

We initially determined whether acute regulation of GSK3 changes the electrical activity of SCN neurons during the day and night using the minimally invasive loose-patch technique^13^. Compared to vehicle, inhibition of GSK3 with a pharmacological inhibitor (CHIR-99021, abbreviated CHIR throughout the manuscript; 1 μM) significantly decreased the SFR of SCN neurons from hypothalamic slices in a phase-specific manner (*P*=0.018; Fig. 1a,b). GSK3 inhibition significantly suppressed SFR during the day between Zeitgeber time (ZT) 4 and 8 (*P*<0.001). Conversely, at night (ZT 14–18), both treatment groups were similarly quiescent (*P*=0.608). The observed day-time suppression was replicated with another GSK3 inhibitor (SB415286; 1 μM), which significantly decreased SFR by 66% compared with vehicle (mean±s.e.m., vehicle: 5.05±0.44; SB415286, 1.71±0.28; *n*=34–38 cells/group). These results suggest that inhibition of GSK3 suppresses SCN neuronal excitability during the day.

The firing rate of neurons can be regulated by numerous mechanisms, including depolarization of the membrane^14^. We conducted whole-cell current clamp recordings to determine whether CHIR-induced suppression of excitability was due to membrane hyperpolarization. Consistent with recent reports^15^,^16^, day-phase SCN neurons exhibited a wide range of resting membrane potentials (RMPs) which was not affected by GSK3 inhibition (*P*=0.133; Fig. 1c). There was also no significant difference in input resistance (Supplementary Fig. 1a,b), suggesting that the decreased excitability in CHIR-treated neurons was not due to an increase in the potassium leak conductance^17^.

As seen in the vehicle-treated controls during the mid-day (Fig. 1c), SCN neurons are capable of exhibiting multiple phenotypes in spontaneous electrical activity^18^, and these characteristics (for example, spiking versus silent) can impact neurophysiological response^19^. Using a k-means cluster analysis based on RMP and event amplitude, we classified each neuron into one of four categories while blind to treatment condition. Based on the results, SCN neurons were classified as either (i) silent, (ii) spiking, (iii) having depolarized electrical states with low-amplitude membrane oscillations or (iv) exhibiting depolarization block, in which spiking could be reinstated by injecting hyperpolarizing current (Supplementary Fig. 2) as reported in other brain areas^20^,^21^ as well as in the SCN^16^. In the remaining spontaneously spiking neurons, APs were separated (or generated) by smooth depolarizing ramps to threshold (Fig. 1e). When GSK3 was inhibited, however, cells within this same voltage range seldom exhibited spontaneous APs (Fig. 1d). Interestingly, some cells displayed low-amplitude membrane potential oscillations. These oscillations were very similar to the effects of the voltage-gated sodium channel blocker, tetrodotoxin (TTX), when applied to SCN neurons^12^,^22^. Given that the frequency of these oscillations was not different from vehicle-treated cells (Supplementary Fig. 1c), we hypothesized that, like TTX, GSK3 inhibition may modulate SCN excitability through suppression of a TTX-sensitive, voltage-gated sodium current. The voltage-gated sodium channels that are responsible for different components of Na^+^ current^23^ are expressed in SCN neurons (Supplementary Fig. 3). While rapidly activating and inactivating transient current (*I*_NaT_) plays a role in the peak of the AP, the persistent Na^+^ current is important in maintaining repetitive firing in many neuron types^24^. We focused our studies on the persistent Na^+^ current (*I*_NaP_), given that it provides excitatory drive to AP threshold in SCN neurons^12^, which appeared to be diminished in CHIR-treated cells. Thus, we next considered the effect of GSK3 inhibition and activation on this current.

### GSK3 inhibition suppresses *I*_NaP_ specifically during the day

**Figure 2:**
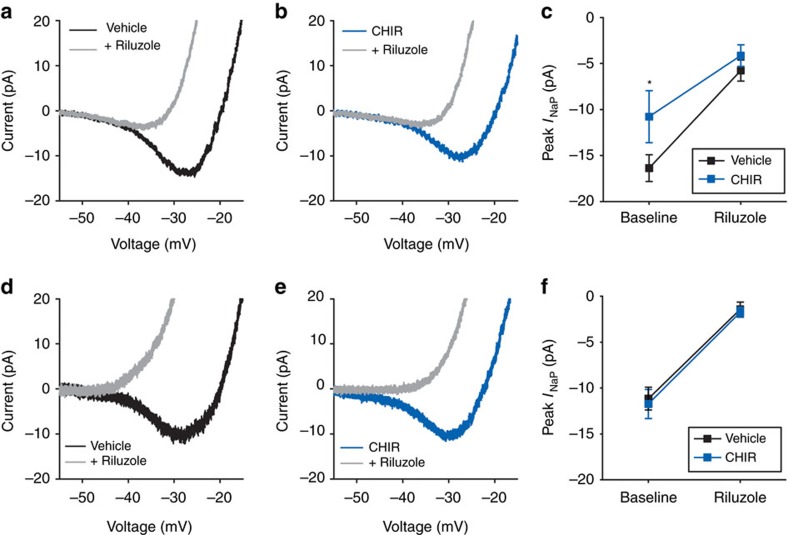
GSK3 inhibition suppresses *I*_NaP_ during the day but not the night. (**a**,**b**,**d**,**e**) Average normalized response to slow depolarizing voltage ramp (−100 to +10 mV; 59 mV s^−1^) from SCN cells treated with (**a**,**d**) vehicle (DMSO, 0.002%; black) or (**b**,**e**) GSK3 inhibitor (CHIR, 1 μM; blue) at baseline and after 3-min treatment with persistent sodium current blocker, riluzole (20 μM; grey). (**c**,**f**) Peak inward current (mean±s.e.m.) at baseline and after riluzole treatment. During the day (**c**) CHIR-treated cells (blue) had significantly reduced inward current at baseline (*P=0.042) that was lost after blockade of *I*_NaP_ with riluzole (*P*=0.350). Two-way, mixed-design ANOVA; Treatment X Riluzole interaction, *F*_(1,31)_=5.455, *P*=0.026; *n*=13–20 cells, four animals per group. At night (**f**) peak inward current did not differ between CHIR- and vehicle-treated cells at baseline or after treatment with riluzole (non-significant main effect of CHIR, *F*_(1,33)_=0.150, *P*=0.748; *n*=13–22 cells, four animals per group). Recordings made between ZT 4–8 (**a**–**c**) or ZT 14–18 (**d**–**f**).

Several sodium channels have been shown to play a role in conducting a slowly inactivating, or persistent, sodium current or *I*_NaP_, in central neurons, including Na_V_ 1.1, 1.2, 1.3 and 1.6 (refs 24, 25), all of which are expressed in the SCN (Supplementary Fig. 3), and at least Na_V_1.6 has been shown to be regulated by GSK3 (refs 26, 27). Although small in magnitude, *I*_NaP_ typically contributes to excitation of repetitively firing neurons by augmenting small depolarizations during the interspike interval^12^. In SCN cells, *I*_NaP_ is proposed to provide the excitatory drive towards threshold^12^, and silencing *I*_NaP_ suppresses AP firing in SCN cells^28^. Therefore, we examined the effect of GSK3 inhibition on *I*_NaP_ by measuring the current response to a slow (59 mV s^−1^), depolarizing voltage ramp from −100 mV to 10 mV (ref. 29) before and after treatment with the selective persistent sodium channel blocker riluzole (20 μM, 3 min). The voltage ramp elicited an inward current in vehicle-treated cells at the range of *I*_NaP_ activation, starting at −52.4±1.1 mV and peaking at −26.8±0.7 mV (as in refs 30, 31, 32; means±s.e.m.; *n*=20 cells). During the day, the magnitude of peak *I*_NaP_ was significantly reduced in CHIR-treated cells compared with vehicle-treated controls at baseline (*P*=0.042; Fig. 2a,b). Riluzole significantly suppressed the amount of inward current (*P*<0.001) and eliminated the difference between CHIR- and vehicle-treated cells (Fig. 2c). However, at night, CHIR did not suppress baseline *I*_NaP_ compared with vehicle-treated controls (Fig. 2d–f). These results suggest that GSK3 inhibition decreases *I*_NaP_ in day-phased SCN neurons and that this current is driven at least in part by GSK3 activation. Because circadian regulation of GSK3 inactivation increases throughout the night when SCN cells are mostly quiescent^5^, GSK3 regulation of *I*_NaP_ provides a previously unexplored mechanism underlying the daily rhythms in SCN excitability.

To investigate whether GSK3 regulates overall sodium current, we next measured the effect of GSK3 inhibition on *I*_NaT_. In the presence of blockers (0.1 mM CdCl_2_, 10 mM TEA, 3 mM 4-AP), the average peak *I*_NaT_ was similar with and without the GSK3 inhibitor (peak *I*_NaT,_ in pA: vehicle, −883.6±109.8; CHIR: −960.4±145.6; *t*_(21)_=0.43, *P*>0.05). While there was no change in the inactivation of *I*_NaT_ (slope factor *V*_C_, vehicle=5.8±1.2; CHIR=5.7±1.1; *t*_(21)_=0.03; *P*>0.05), GSK3 inhibition positively shifted the activation of this current by ∼8 mV (vehicle: *V*_mid_=−29.3±1.7 mV; CHIR: *V*_mid_=−20.7±2.0 mV; *t*_(21)_=3.3, *P*<0.05). Thus, GSK3 inhibition did not appear to affect INaT magnitude but may impact regulatory sodium channel subunits, such as one of the four β subunits^33^.

### *I*_NaP_ is regulated by time-of-day and activation of GSK3

Complementary to the present results that GSK3 inhibition suppressed SCN activity during the day but not the night, we have recently reported that chronic GSK3 activation increases neuronal firing at night, but not the day^4^. To determine if chronic GSK3 activation enhances *I*_NaP_ in SCN neurons, we measured *I*_NaP_ currents from wild-type (WT) mice and transgenic mice with constitutive GSK3 activation (that is, GSK3-KI mice) due to serine–alanine substitutions at the two inhibitory phosphorylation sites in subunits GSK3α and GSK3β (S21 and S9, respectively)^34^. Although previous reports have suggested that *I*_NaP_ does not exhibit a day/night difference in the SCN based on transcriptional gene expression^3^,^35^; until now, no study has examined *I*_NaP_ isolated current at both times of the day.

**Figure 3:**
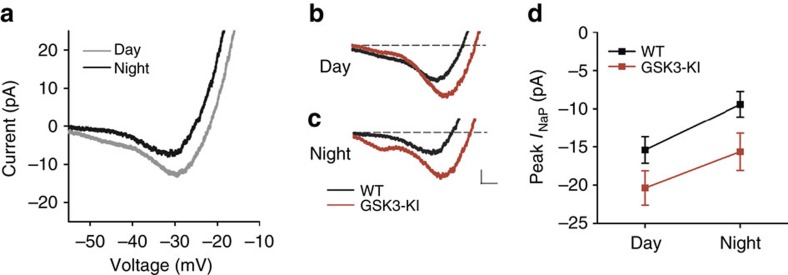
*I*_NaP_ enhanced during the day and by chronic GSK3 activation. (**a**) Average normalized response to slow depolarizing voltage ramp (−100 to +10 mV; 59 mV s^−1^) from SCN cells from WT during the mid-day (grey) or early-night (black). (**b**,**c**) Average normalized responses to voltage ramp from WT (black) or GSK3-KI (red) SCN cells during the day (**b**) or night (**c**). Scale bar, 5 pA, 5 mV. (**d**) Means±s.e.m. of peak inward current from cells in (**a**–**c**). Two-way ANOVA; main effect of time, *F*_(1,80)_=7.009, *P*=0.01; main effect of genotype, *F*_(1,80)_=7.506, *P*=0.008. For all panels: WT day, *n*=28 cells, five animals; WT night, *n*=22 cells, three animals; GSK3-KI day, *n*=18 cells, four animals; GSK3-KI night, *n*=16 cells, 3 animals.

We found that WT neurons exhibited a significant increase in *I*_NaP_ during the day compared with the night (*P*=0.02; Fig. 3a). Further, *I*_NaP_ was enhanced in GSK3-KI cells compared with WT cells (*P*=0.008; Fig. 3b,c). Surprisingly, the effect of chronic GSK3 activation was independent of time of day as both genotypes exhibited a day–night difference in *I*_NaP_ magnitude (*P*=0.01; Fig. 3b–d). Follow-up recordings from WT and GSK3-KI cells at night in the presence of CdCl_2_ (0.1 mM) and TEA (10 mM) yielded similar results, yet this current could be blocked by a different Na^+^ channel blocker, TTX (Supplementary Fig. 4). These results suggested that this TTX-sensitive, inward current was not driven by calcium or potassium currents and could be due to shuttling or sequestration of constitutively active GSK3.

### Mathematical model predicts GSK3 effect on *I*_NaP_

To investigate how GSK3 affects neuronal excitability through *I*_NaP_, we incorporated the *I*_NaP_ current into a conductance-based single-cell model of SCN electrophysiology that simulates electrical activity with millisecond resolution based on sodium, potassium, calcium (L-type and non L-type), calcium-activated potassium and leak (Na-leak and K-leak) currents^17^. We modified the published model with the addition of *I*_NaP_ with dynamics fit using the voltage ramp data (Figs 2 and 3) as described in the Methods section. We found that the dynamics of *I*_NaP_ are consistent with a conductance-based model of the form *I*_NaP_=−*g*_NaP_(*V*−*E*_Na_)*p*, with the activating gating variable *p* ranging between 0 and 1. Currents for all experimental conditions could be fit to this form by varying the maximal conductance *g*_NaP_ (Supplementary Fig. 5; *g*_NaP_ values are GSK3-KI, day: 2.27; GSK3-KI, night: 2.13; WT, day: 2.09; WT, night: 1.59; CHIR, day: 1.97; CHIR, night: 1.46). This finding suggests that the action of GSK3 on *I*_NaP_ is primarily through changing the maximal conductance of *I*_NaP_ channels rather than the channel kinetics.

**Figure 4:**
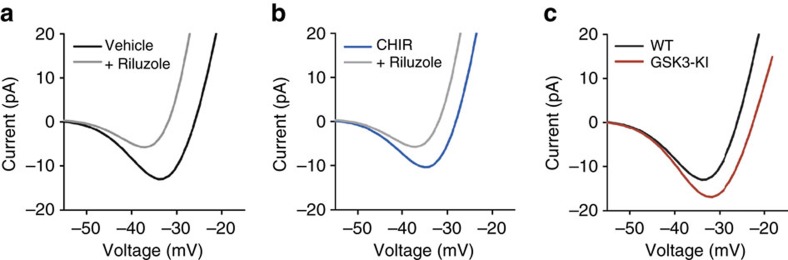
Modelling of voltage-ramp experiments. (**a**) Simulations of the voltage-ramp protocol in WT model cells with and without riluzole (*R*=3.1, vehicle: *g*_NaP_=2.08, riluzole: *g*_NaP_=0). Our model matched the data collected in Fig. 2a and shows the effects of the persistent sodium current in the model. (**b**) Simulations of the voltage-ramp protocol under CHIR with and without riluzole (*R*=3.1, vehicle: *g*_NaP_=1.46, riluzole: *g*_NaP_=0). This matches Fig. 2b and shows the effect of inhibiting GSK3 in our model. (**c**) Simulations of the voltage-ramp protocol in the GSK3-KI compared with WT (*R*=3.1, WT: *g*_NaP_=2.08, GSK3-KI: *g*_NaP_=2.85). This matches the data in Fig. 3b, and shows how the overactive kinase increases the persistent sodium current.

We validated the model after the addition of the *I*_NaP_ current by performing voltage ramp simulations mimicking the analogous experiments presented in Figs 2 and 3 (Fig. 4). Simulated voltage ramps with and without *I*_NaP_ matched the magnitudes of the inward currents seen experimentally in each condition: with simulated application of vehicle (Fig. 4a), CHIR (Fig. 4b), and with simulated constitutively active GSK3 (Fig. 4c), where each condition is reproduced simply by tuning the maximal conductance of the *I*_NaP_ current (*g*_NaP_).

### *I*_NaP_ mediates GSK3-induced excitability at night

**Figure 5:**
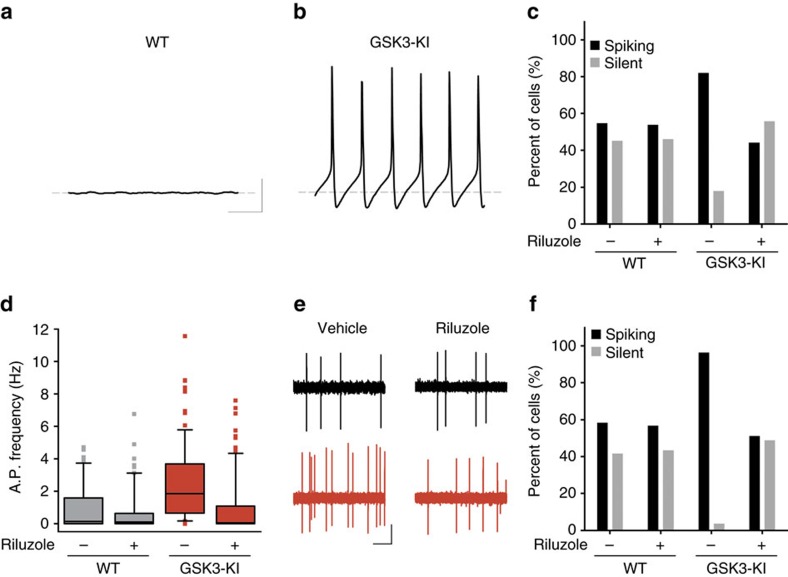
Riluzole blocks GSK3-induced excitability. (**a**,**b**) Representative model recordings (1 s) from nighttime cell with *I*_NaP_ at WT (**a**) or GSK3-KI (**b**) level shows that increasing *I*_NaP_ alone is sufficient to induce spontaneous activity in a silent cell. Scale bar, 20 mV, 200 ms. Grey line indicates RMP (−63.4 mV). (**c**) Percentage of silent versus spiking neurons seen in SCN network model at night with WT or GSK3-KI levels of *I*_NaP_ before and after *I*_NaP_ blockade with riluzole. (**d**) Box plot of early-night spontaneous AP frequencies of SCN neurons from WT (grey) or GSK3-KI (red) mice treated with vehicle (DMSO, 0.01%) or riluzole (10 μM), showing 10th and 90th percentiles (whiskers), 25th and 75th percentiles (box borders), median (centre line) and outliers (symbols). Scheirer–Ray–Hare Kruskal–Wallis test, main effect of genotype, *H*_(1)_=7.009, *P*<0.001; main effect of treatment, *H*_(1)_=8.089, *P*<0.001 and interaction, *H*_(1)_=4.834, *P*<0.001, *post hoc* asymptotic significance, *P*<0.001. (**e**) Representative cell-attached loose-patch traces (5 s) from each group. Scale bar, 20 mV, 1 s. (**f**) Quantification of silent versus non-silent cells for each group in **d** and **e**. Three-way loglinear analysis, three-way interaction, *χ*^2^_(1)_=25.852, *P*<0.001. Follow-up *χ*^2^ tests revealed that GSK3-KI cells were significantly more likely to be spiking than WT vehicle-treated cells (*χ*^2^_(1)_=32.428, *P*<0.001). Blocking *I*_NaP_ with riluzole increased the proportion of silent cells in GSK3-KI slices (*χ*^2^_(1)_=44.735, *P*<0.001) up to that of WT levels (*χ*^2^_(1)_=0.430, *P*=0.313), whereas riluzole had no effect on the proportion of silent cells in WT slices (*χ*^2^_(1)_=0.034, *P*=0.5). For **d** and **f**: WT and WT+riluzole, *n*=60 cells, 2 animals; GSK3-KI, *n*=84 cells, three animals; GSK3-KI+riluzole, *n*=86 cells, three animals.

Since the effects of *I*_NaP_ have been shown to be different in isolated cells or in a network, we incorporated this ionic single-cell model into a previously validated SCN neuronal network model^18^,^36^. This model also included a validated model of the intracellular timekeeping system within individual cells. A total of 1,024 γ-aminobutyric acid-ergic (GABAergic) and vasoactive intestinal polypeptide-coupled SCN neurons were studied. Neurons within the network were excited or inhibited by GABA depending on cellular GABA reversal potentials, which had a distribution based on previously published experimental measurements^36^. This led to a distribution of excitation across the SCN causing WT cells to exhibit the varied firing activities reported here and in previous studies with circadian variation^36^. Using the model, we tuned the strength of *I*_NaP_ to mimic the transition from low to normal to high GSK3 activity (for example, CHIR to WT to GSK3-KI) and then examined the resulting changes in neuronal excitability. As *g*_NaP_ is increased to the level of GSK3-KI mice, the model predicted that many of the cells which were normally quiescent at night became spontaneously active. Voltage for a sample quiescent WT neuron at night varied little from the RMP (Fig. 5a), whereas the identical simulated neuron with *g*_NaP_ increased to GSK3-KI levels fired APs spontaneously (Fig. 5b). Furthermore, approximately half of cells in the WT network were quiescent at night, while in the GSK3-KI network, the majority of neurons were spiking (Fig. 5c). Since all parameters other than *g*_NaP_ were identical between the simulations, the change in activity must have been due to the persistent sodium current.

To test this model prediction, we examined the SFR of WT and GSK3-KI SCN neurons following application of riluzole (10 μM) and compared it to vehicle controls. Consistent with our previous work, vehicle-treated GSK3-KI cells had a significantly increased SFR compared with WT neurons (*P*<0.001; Fig. 5d,e). This hyper-excitability was rescued in riluzole-treated GSK3-KI cells (*P*<0.001), which did not differ from either WT group. Since the majority of SCN neurons are relatively quiescent during the night, we also quantified the number of spiking versus non-spiking cells from WT and GSK3-KI mice in riluzole- and vehicle-treated conditions and found significant differences between groups (*P*<0.001; Fig. 5f). While ∼40% of WT neurons were silent, almost all of the GSK3-KI neurons were spiking (*P*<0.001), as predicted by the model. Moreover, the ratio of spiking to non-spiking cells was restored to WT levels upon riluzole treatment, while riluzole had no effect on WT neurons at night (*P*=0.5; Fig. 5f). Taken together, these results suggest that active GSK3 promotes neuronal excitability in an *I*_NaP_-dependent manner.

### GSK3 promotes excitability through AHP but not RMP

**Figure 6:**
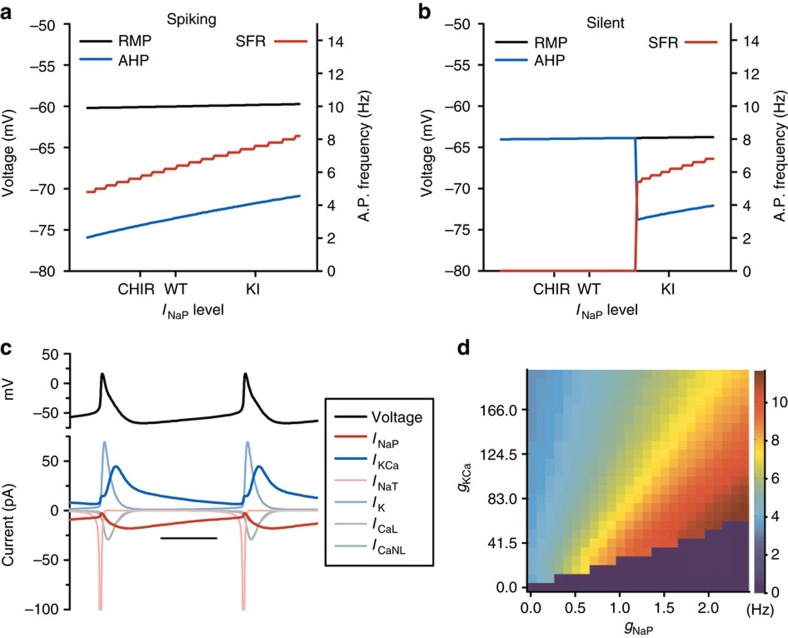
Model predicts *I*_NaP_ promotes excitability through AHP but not RMP. (**a**,**b**) Model prediction of RMP (black), minimum voltage attained after an AP (AHP; blue) and SFR (red) at different levels of *I*_NaP_ for a WT-spiking (**a**) or WT-silent (**b**) SCN neuron at night. The WT-silent neuron does not spike with low to moderate levels of *I*_NAP_, and has a SFR of zero and no AHP. When *g*_NaP_ is increased past a threshold level, the cell undergoes a ‘bifurcation' in its dynamics and begins to fire. When either neuron fires, increasing *I*_NaP_ increases the SFR without changing the RMP by decreasing the magnitude of the AHP. (**c**) Various currents (colour-coded in legend) associated with a typical SCN AP. During the AHP and ramp back to threshold, all currents other than *I*_NaP_ and *I*_KCa_ have inactivated. Scale bar, 40 ms. (**d**) Heat map showing the contributions of *g*_NaP_ and *g*_KCa_ towards setting the SFR (in Hz) of SCN neurons.

We next investigated how *I*_NaP_ affects intrinsic excitability. Often, increases in excitability are due to depolarization of the RMP; however, the model unexpectedly predicted that increasing *I*_NaP_ in firing neurons causes an increase in firing rate with minimal change to the RMP of the cells (Fig. 6a,b). This model prediction is consistent with our experimental results that GSK3 inhibition suppresses SCN activity without affecting RMP (Fig. 1).

Further analysis of our model simulations revealed that the increased excitation through *I*_NaP_ is instead caused by suppression of the AP afterhyperpolarization (AHP). In both spiking and initially silent WT neurons at night, increasing the magnitude of *I*_NaP_ by increasing *g*_NaP_ from CHIR to GSK3-KI levels caused the minimum voltage attained after an AP to become more depolarized without changing the RMP (Fig. 6a,b). Thus the magnitude of the AHP diminished, allowing cells to fire more rapidly (Fig. 6a,b). Note that in the WT-silent neuron (Fig. 6b), increasing *g*_NaP_ beyond a threshold level caused a bifurcation in the dynamics of the cell, and the onset of oscillations^37^, as seen in Fig. 5. According to the model, the degree of AHP following an AP depends on the balance of *I*_NaP_ (positive-inward) and *I*_KCa_ (positive-outward) currents. By plotting each of the currents that contribute to changes in membrane voltage, it is apparent that most of the currents involved in generating the AP shut off rapidly (Fig. 6c). Only *I*_NaP_ and *I*_KCa_ persist through the interspike interval, setting the AHP magnitude and the depolarizing ramp back towards threshold. Increasing *I*_NaP_ or decreasing *I*_KCa_ causes a decrease in the AHP magnitude and consequently speeds up the firing rate. Conversely, decreasing *I*_NaP_ or increasing *I*_KCa_ increases the AHP magnitude and slows firing (Fig. 6d).

**Figure 7:**
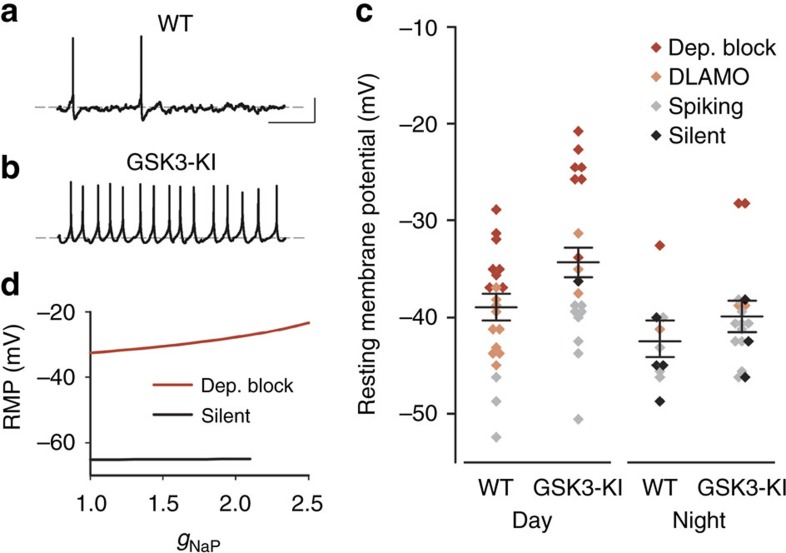
GSK3 activity promotes nighttime excitability without affecting RMP. (**a**,**b**) Representative whole-cell current clamp recordings (5 s) from WT (**a**) and GSK3-KI (**b**) SCN neurons in early-night. Scale bar, 20 mV, 1 s. Dashed grey line indicates −40 mV. (**c**) Dot plot of RMP for individual WT or GSK3-KI SCN neurons recorded from during the day or early-night. Lines indicate mean±s.e.m. for each group. Cells were significantly more depolarized during the day and in GSK3-KI slices. Two-way ANOVA; main effect of time, *F*_(1,64)_=7.474, *P*=0.008; main effect of genotype, *F*_(1,64)_=6.099, *P*=0.016. From left-to-right, *n*=21 cells, four animals; 19 cells, three animals; 11 cells, three animals; 17 cells and three animals. The effect of genotype on RMP was driven by groups of cells in depolarized block (non-significant main effect of genotype when depolarized block cells were excluded from analysis; *F*_(1,54)_=0.623, *P*=0.433). Symbol colour indicates subgroup phenotype as indicated in the legend. (**d**) Model prediction of effect of *I*_NaP_ conductance (*g*_NaP_) on RMP shows that increasing *g*_NaP_ causes a cell in depolarization block (red) to further depolarize but has no effect on the RMP of a hyperpolarized, silent cell (black).

**Figure 8:**
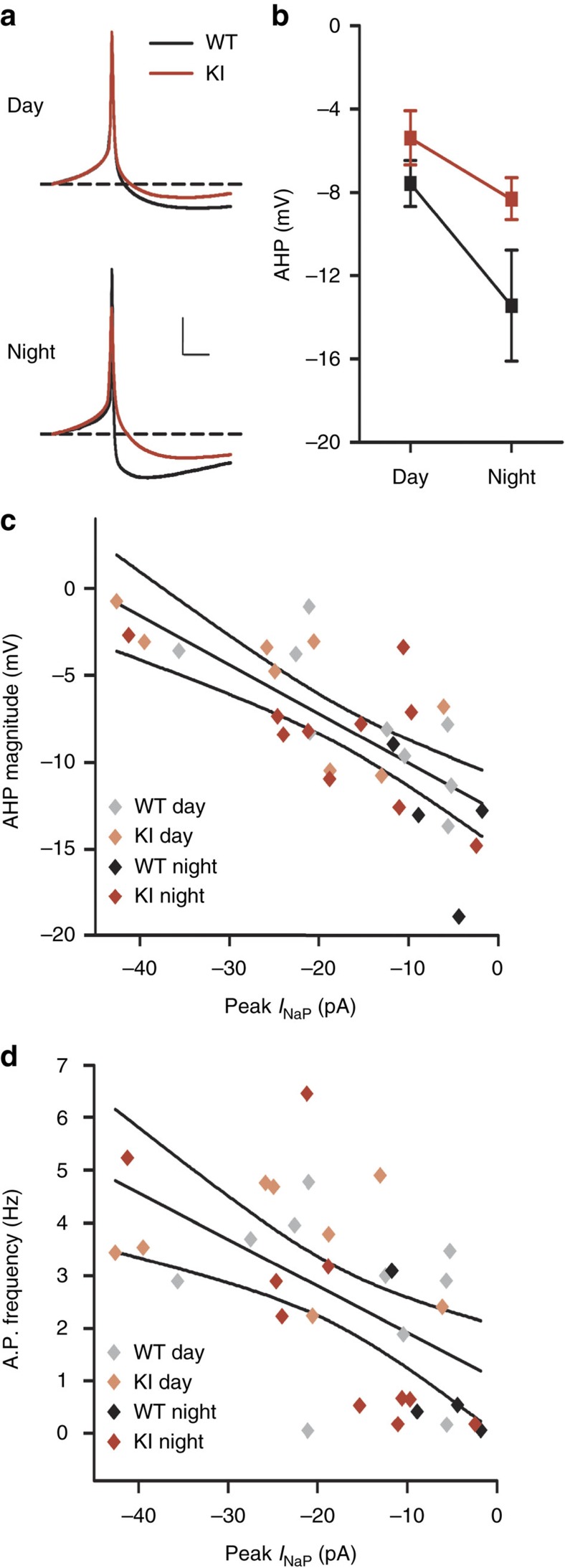
GSK3 decreases the AHP magnitude through *I*_NaP_. (**a**) Average AP waveforms from spontaneously active SCN neurons from WT (black) or GSK3-KI (red) mice recorded in mid-day or early-night. For visualization, all waveforms were adjusted to same baseline (−40 mV, dashed line) before averaging. Scale bar, 10 mV, 10 ms (**b**) Mean±s.e.m. of AHP magnitude (difference from RMP) from cells represented in **a**. AHP amplitude was significantly decreased during the day and in GSK3-KI cells. Two-way ANOVA; main effect of time, *F*_(1,30)_=10.266, *P*=0.003; main effect of genotype, *F*_(1,30)_=7.085, *P*=0.012. For **a** and **b**: *n*=4–11 cells, three animals per group. (**c**) Amplitude of AHP and peak *I*_NaP_ of spiking cells from all groups are significantly correlated. Pearson correlation, *R*=−0.732, *P*<0.001. Symbol colour indicates group as indicated in the legend. Lines represent linear fit and 95% confidence intervals of all cells in plot. (**d**) Spontaneous AP frequency and peak *I*_NaP_ of spiking cells from all groups are significantly correlated. Pearson correlation, *R*=−0.550, *P*=0.001. Lines represent linear fit and 95% confidence intervals for all cells in plot. For **c** and **d**: *n*=4–10 cells per group.

To test the prediction that increasing *I*_NaP_ decreases AHP without affecting RMP experimentally, we performed whole-cell current clamp recordings in WT and GSK3-KI neurons during the day and night (Fig. 7a,b). As predicted by the model, there was no correlation between peak *I*_NaP_ and RMP (*r*=−0.094, *P*=0.463; Supplementary Fig. 6). Both genotypes exhibited a day/night difference in RMP (*P*=0.008), and there was no difference in RMP between genotypes for the majority of neurons (two-way analysis of variance (ANOVA), with depolarization block cells excluded; *P*=0.433; Fig. 7c). Only when the subset of neurons in depolarization block were included in the analysis did constitutive activation of GSK3 result in a more depolarized RMP (two-way ANOVA, with all cells included; *P*=0.016; Fig. 7c). This effect was predicted by the model as well since the voltage-gated sodium channels responsible for *I*_NaP_ are held open at the higher voltages seen in depolarization block-type neurons, leading to an increase in RMP (Fig. 7d). Additionally, analysis of the AP waveforms of the spontaneously active cells from the same recordings revealed a significant decrease in the AHP in the GSK3-KI neurons (*P*=0.012), particularly during the night, such that the day–night difference in AHP is diminished in GSK3-KI neurons (Fig. 8a,b). Moreover, consistent with the model prediction, both the AHP amplitude and the SFR of all spontaneously spiking cells were significantly negatively correlated with *I*_NaP_ peak current, such that the larger the *I*_NaP_ magnitude, the smaller the AHP (*r*=−0.732, *P*<0.001; Fig. 8c) and faster the SFR (r=−0.550, *P*=0.001; Fig. 8d). To directly test whether *I*_NaP_ magnitude influences AHP, a follow-up current clamp experiment was conducted to measure the AHP of individual SCN neurons before and after acute application of riluzole (10 μM). As shown in Supplementary Fig. 6b,c, blockade of *I*_NaP_ significantly induced a 70% increase in AHP amplitude (*n*=5; mean change±s.e.m.: 1.66±0.63 mV; *P*=0.001), but had no effect on RMP (mean change±s.e.m.: −0.23±0.42 mV; *P*=0.605).

## Discussion

Here, we present evidence that the rhythmically phosphorylated kinase GSK3 (ref. 5) promotes neuronal excitability through regulation of *I*_NaP_. In particular, we find that: (1) GSK3 activity regulates *I*_NaP_, (2) *I*_NaP_ exhibits a day/night difference in peak magnitude that is regulated by GSK3 and (3) *I*_NaP_ promotes firing and decreases AP AHP without affecting RMP. Our experiments were conducted in the SCN, the site of the central circadian pacemaker. As molecular circadian timekeeping can be found in many parts of the brain^38^, this mechanism could be widely used in the brain to control many systems. Of particular interest are the regions of the brain that regulate mood. Impaired molecular circadian timekeeping has been implicated in mood disorders^39^, and differentiated neurons from induced pluripotent stem cells isolated from patients with bipolar disorder show enhanced SFRs and over-excitability^40^. Given that GSK3 phosphorylation and *I*_NaP_ are altered by mood stabilizing drugs such as lithium^41^ and riluzole^42^, respectively, this work may provide a potential basis for the chronotherapeutic control of mood.

Despite the known importance of *I*_NaP_ as a pacemaker potential in many types of spontaneously active neurons^43^,^44^,^45^,^46^, including the SCN^12^,^28^,^29^, *I*_NaP_ has been largely overlooked as a potential contributor to the circadian modulation of rhythmic firing^3^,^28^,^47^. However, the present study is the first to show that *I*_NaP_ is greater in magnitude during the day (∼5 pA) than at night. This difference in *I*_NaP_ magnitude is sufficient to have a notable impact on excitability of compact, high R_input_ neurons such as those in the SCN. Prior work has shown that blockade of *I*_NaP_ with riluzole acutely silences spontaneous firing^28^ and chronically dampens the circadian amplitude of SFR rhythms of individual SCN neurons^47^. Conversely, our results demonstrate that high, day-like *I*_NaP_ levels seen in GSK3-KI neurons at night are sufficient to induce spiking in neurons that would normally be silent (Fig. 6) as well as increase the SFR of already spiking neurons (Fig. 7a). Moreover, lower *I*_NaP_ level following GSK3 inhibition reduces the SFR (Figs 1 and 2), while transient sodium current was minimally affected.

Although GSK3 may impact neurophysiological properties of neurons through regulation of other ionic conductances yet to be identified (including *I*_KCa_), our results show a novel effect of GSK3 activity on excitability through modulation of *I*_NaP_. This GSK3-mediated increase in *I*_NaP_ may be the result of several different channel protein modifications including channel trafficking, distribution and phosphorylation state^3^. In fact, recent work in the hippocampus has shown that fibroblast growth factor 14 forms a complex with at least one sodium channel alpha subunit (that is, Na_V_1.6) in a GSK3-dependent manner^26^. It is interesting to note that four of the alpha subunits implicated in *I*_NaP_ are expressed in the SCN (Supplementary Fig. 3), with enrichment specifically for Na_V_1.3. In addition to alpha subunits, GSK3 may also activate other regulatory subunits of voltage-gated sodium channels, such as the Na_V_ β subunits, which can enhance *I*_NaP_ magnitude^33^. Future research will be necessary to elucidate the contribution of the various TTX-sensitive Na_V_ channel subunits responsible for day–night differences in *I*_NaP_ magnitude in SCN neurons.

Our work also shows how changes in *I*_NaP_ permeate throughout the SCN network. Since the SCN is coupled via mainly inhibitory GABA, increasing the firing rate causes more inhibitory post-synaptic currents, which could slow firing. However, inhibitory post-synaptic currents can both increase and decrease firing in SCN neurons^36^, so the effects of inhibitory GABA are not always intuitive. Nevertheless, our modelling and experimental data show an increase in firing rate throughout the network, and in particular, a change in the electrical state of neurons that produces an increase in the proportion of neurons that are firing. Together, this work suggests GSK3 as a way of modulating not just firing of a single cell, but also global network properties. Future work can improve our mathematical models by considering the morphology of SCN neurons, additional neurotransmitters and neuropeptides besides GABA and the stochasticity of neuronal firing.

Further clarification of the details of GSK3 regulation is an interesting question for future study. Despite much work, how the circadian clock precisely regulates GSK3 activity is unknown. In addition to rhythmic inhibitory phosphorylation, GSK3 function within the SCN could also be reduced by sequestration, localization or through priming kinases^48^. Although suppression of *I*_NaP_ by pharmacological GSK3 inhibition was time-of-day specific (that is, restricted to the day, when GSK3 activity is high), GSK3-KI mice with chronic GSK3 de-phosphorylation had enhanced, but unexpectedly rhythmic *I*_NaP_, suggesting that other mechanisms of GSK3 inhibition may still be rhythmic in these mice. Alternatively, we cannot rule out a compensatory mechanism or developmental effect as an explanation of this result; however, it should be noted that no gross developmental differences have been previously reported in this animal model^34^.

By combining modelling and experimental approaches, we present an intriguing method by which the molecular circadian clock controls electrical activity. Unlike Na^+^ leak currents which were recently shown to promote excitability of clock neurons by membrane depolarization^49^, our data show a mechanism for how *I*_NaP_ regulates the firing rate of APs without affecting the RMP of neurons that are hyperpolarized or spiking. We also show that the balance between *I*_NaP_ and *I*_KCa_ controls both the depolarizing ramp towards the AP threshold and, surprisingly, the magnitude of the AHP following an AP. In fact, pharmacological inhibition of *I*_NaP_ increased AHP magnitude without affecting RMP, consistent with a prior study that showed that reduction of *I*_NaP_ due to genetic loss of SCN8A significantly increases peak AHP in cerebellar granule cells^50^. Thus, sub-threshold ionic balance could be crucial to rhythmic changes in AP frequency independent of circadian phase, especially in light of a previous modelling/experimental study which showed that changes in RMP can feedback to phase shift the circadian clock^36^. The mechanism we propose, therefore, can faithfully reflect the state of the circadian clock in firing rate without disrupting molecular circadian timekeeping itself.

Intriguingly, aging-induced circadian decline is associated with reduced SCN firing rate amplitude and smaller nocturnal AHP in the absence of altered PER2 expression^51^,^52^, leading to the proposal that SCN neuronal activity may be the ‘weak link' in the circadian system^3^. Given that p-GSK3 levels are decreased in the SCN of aged rodents, GSK3 control of *I*_NaP_ may be one source of age-related circadian disruption. While more work is necessary to explore this concept as well as the biophysical mechanism by which GSK3 activates *I*_NaP_, this work suggests a promising new motif in which a kinase can directly regulate electrical activity acutely and without transcriptional cost. Because of its efficiency, this is a motif likely to be found in other brain regions as well.

## Methods

### Animals

Homozygous *Per1*::GFP^53^, WT C57-BL6/J, and GSK3α/β^21A/21A/9A/9A^ (GSK3-KI) mice (2–5 months old; male and female) backcrossed for at least 10 generations to C57BL/6 mice, in which regulatory serine–alanine substitution on both isoforms of GSK3 rendered GSK3 de-phosphorylated and constitutively active^34^ were group-housed (2–5 animals per cage) in a 12:12 light/dark cycle with *ad libitum* access to food and water, and handled in accordance with the University of Alabama at Birmingham (UAB) Institutional Animal Care and Use Committee (IACUC) and National Institutes of Health (NIH) guidelines. Mice were euthanized with cervical dislocation and rapid decapitation.

### Electrophysiology

All chemicals were obtained from Sigma-Aldrich (St Louis, MO) unless otherwise specified. For recordings in which the primary outcome was AP firing rate, the loose-patch recording technique was chosen, given that it does not affect endogenous membrane properties either by formation of a tight seal or by dialysis of intracellular milieu^13^,^54^,^55^. Sample sizes were based on previous work with similar effect sizes and significant differences between groups, given the same methodologies for both loose patch and whole-cell electrophysiology^56^,^57^,^58^. Fresh brain slices were prepared from mice killed between ZT 2–3 or ZT 11–12 for day and night recordings, respectively, using previously published methods (as in ref. 4). All recordings were made between ZT 4–8 and ZT 14–18. For GSK3 inhibitor experiments, the chamber was perfused with extracellular solution (in mM: 130 NaCl, 20 NaHCO_3_, 1 Na_2_PO_4_-7H_2_O, 1.3 MgSO_4_-7H_2_O, 10 glucose, 3.5 KCl, 2.5 CaCl_2_) containing 1 μM CHIR-99021 (Stemgent, San Diego, CA) or vehicle (0.002% DMSO) starting at ZT 3.5 or ZT 13.5, and targeted recordings were made from SCN cells between ZT 4–8 and 14–18. For riluzole loose-patch recordings, bath-application of vehicle (0.01% DMSO) or 10 μM riluzole began at ZT 13 and recordings were made from ZT 14–18. Electrodes with a pipette resistance of ∼4–6 MΩ were filled with filtered, K^+^-gluconate solution (as in ref. 4; in mM: 135 K-gluconate, 10 KCl, 10 HEPES, 0.5 EGTA; pH 7.4). Firing rate was measured as the average of a 120-s record. Comparisons of genotypes did not allow randomization to WT/GSK3-KI groups (but see congenic strain background above). In addition, no specific methods were used to randomize mice to experimental groups or to blind investigators to treatment; however, treatment, time-of-day and genotype measurements were always interleaved.

### Whole-cell electrophysiology

Slices were prepared using the same methods and solutions described previously^4^ and above. Riluzole-sensitive current was measured using a slow depolarizing voltage ramp (−100 mV to +10 mV, 59 mV s^−1^) before and after 3 min riluzole (20 μM) application^28^. This ramp speed was chosen because it was in the middle of the range used by prior studies in SCN neurons^12^,^28^. Moreover, this ramp speed produced maximal current without compromising voltage control (similar to ref. 29), and the same ramp speed was used for all experiments. At least 5–10 sweeps of the voltage ramp protocol were averaged for each cell, and baseline subtracted by fitting the linear portion between −85 mV and −65 mV to zero^28^. Peak sodium current was determined as the minimum point of a 4–7th order polynomial fit applied to the baseline subtracted curve between −55 mV and −10 mV. For current clamp recordings, cells were recorded in gap-free current clamp mode. RMP was determined as the average voltage half-way between two APs. Cells were classified by neurophysiological phenotypes based on RMP and spontaneous event amplitude. Using a k-means cluster analysis, each neuron was grouped into one of four categories while blind to experimental group. Based on the results, SCN neurons were classified as either (1) silent, (2) spiking, (3) having depolarized electrical states with low-amplitude membrane oscillations or (4) exhibiting depolarization block^18^. For cells classified as spiking, the AP AHP was measured using the template search function in Clampfit 10 (Axon Instruments) to determine the average anti-peak amplitude for each cell. In all whole-cell experiments, recordings were made within 5 min of breaking into the cell. Cells with >−50 pA of leak recorded in the seal test, with excessive break through spikes in voltage-ramp protocol (indicative of poor voltage control), or which did not exhibit a rebound spike after terminating a hyperpolarizing current injection were excluded from the analysis. Supplementary Figure 2 depicts an example recording from a depolarized block neuron that was induced to spike with hyperpolarizing current.

### Quantitative, real-time RT-PCR

Slices (600 μm thick) containing the SCN were sectioned in Hanks' balanced salt solution (14175-103; Gibco, Carlsbad, CA) supplemented with NaHCO_3_ (7.5%; Sigma, St Louis, MO), and 1.0 M HEPES according to previously described methods^4^,^57^. The SCN was carefully dissected from the slice under a Zeiss dissection microscope, and tubes containing either the SCN or the remaining brain tissue from the slice were rapidly frozen in liquid nitrogen in RNAase-free tubes and stored at −80 °C until use. RNA isolation and transcript measurements were performed as described in ref. 59. Samples were homogenized using an Omni Bead Ruptor Homogenizer (OMNI International, Kennesaw, GA, USA). Following homogenization, samples from one male and one female WT mouse were combined to give a total of six samples for the SCN and for whole brain. RNA was isolated using the Trizol/chloroform-isopropanol method (Invitrogen, Carlsbad, CA, USA) per manufacturer's protocol and purity and concentration were determined using a Thermo Scientific NanoDrop2000 (Fisher Scientific, Pittsburg, PA, USA). Equivalent amounts of RNA (1 mg) were treated with DNase I (Promega, Madison, WI, USA) at 37 °C for 30 min followed by DNase Stop solution at 65 °C for 15 min and reverse-transcribed using the High-Capacity cDNA Archive Kit (Applied Biosystems, Carlsbad, CA, USA). Transcript measurement was performed using JumpStart Taq Readymix (Sigma) and Applied Biosystems primers with an initial ramp time of 2 min at 50 °C and 10 min at 95 °C and 40 subsequent cycles of 15 s at 95 °C and 1 min at 60 °C. Relative concentrations of the genes of interest were calculated in comparison to a standard curve generated from a doubled concentration as well as 1:5, 1:10, 1:20 and 1:40 dilutions of a pooled cDNA of whole-brain samples (that is, the calibrator method). Taqman primer/probe information is included in Supplementary Table 1 for the following transcripts: *β-actin*, *Scn1a*, *Scn1b*, *Scn2a1*, *Scn2b*, *Scn3a*, *Scn3b*, Scn4b and *Scn8a*. Values were normalized to β-actin and expressed as mean+s.e.m.

### Data analysis

Data were analysed with independent samples *t*-tests, two-way ANOVAs, linear mixed model two-way ANOVA, Pearson's correlation, three-way loglinear analysis and *χ*^2^ tests with SPSS 21.0 (IBM Statistics). In the event that assumptions of normality and homogenous variances were not met (tested by Shapiro–Wilks and Levene's tests, respectively), a nonparametric Kruskal–Wallis or the Scheirer, Ray and Hare extension of the Kruskal–Wallis was used instead. Significance was ascribed at *P*<0.05.

### Model fitting of *I*_NaP_

The voltage dependence of *I*_NaP_ was determined using the current response to a 59 mV s^−1^ depolarizing voltage ramp, from −100 mV to 10 mV. Current values were fit for voltages between −65 mV and −20 mV, since cellular voltages are usually in this range. For fitting, the transient sodium current in the model was ignored since the effects of this current were seen in some experimental recordings, but not all, and never contributed to the inward current in the same voltage range as *I*_NaP_. The least current was seen in night phase cells after treatment with riluzole. This was taken as the baseline, and subtracted away from current response curves for day and night phase cells in three experimental conditions: WT, after treatment with the GSK3 blocker CHIR, and in slices from GSK3-KI animals. The dynamics of the *I*_NaP_ current were consistent with a conductance-based model of the form *I*_NaP_=−*g*_NaP_(*V*−*E*_Na_)*p*, with the activating gating variable *p* ranging between 0 and 1. Fitting the currents, we found that *p* should satisfy the standard Hodgkin–Huxley style gating variable equation


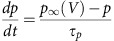


with


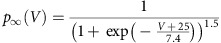


and *τ*_P_=100. Currents for all experimental conditions could be fit to this form by varying *g*_NaP_ (Supplementary Fig. 5: *g*_NaP_ values are GSK3-KI day 2.27, GSK3-KI night 2.13, WT day 2.09, WT night 1.59, CHIR day 1.97 and CHIR night 1.46).

### Circadian network model

The fit *I*_NaP_ current was included into a detailed multiscale ordinary differential equation model of the SCN^36^ containing both a molecular clock model, describing the transcriptional/translational feedback loops at the heart of circadian timekeeping, as well as detailed cellular electrophysiology with millisecond resolution. The SCN electrophysiology model was adapted from ref. 18 and included circadian variation in two conductances (*g*_KCa_ and *g*_K-leak_). We additionally incorporate the *I*_NaP_ current and GSK3 control of the *I*_NaP_ conductance through the function *S*=21.0*(gto-1.66), where gto is the molecular clock variable corresponding to GSK3 activity^60^,^61^. The maximal *I*_NaP_ conductance is then given by:


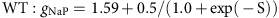



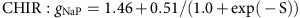



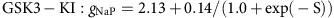


according to the values for day and night in each experimental condition, as in Supplementary Fig. 4. This reproduces the day–night difference in *I*_NaP_ amplitude described above. To compensate for the addition of this current, we reduce the circadian drive of *g*_KCa_ and *g*_K-leak_ to 60% of its original value (*R*=6.81*clk*(G-0.25)). Additionally, the magnitude of the GABA current was reduced (*g*_GABA_=0.1). All other parameters including intercellular signalling and network connectivity were as in ref. 36. For network simulations, the experimentally measured chloride distribution was used to determine GABA reversal potentials as in Fig. 3 of ref. 36. With these small modifications, the model reproduces the circadian variation in electrical activity seen experimentally and the many electrical states seen within the normal SCN 24-hour cycle (Fig. 5c and data not shown)^16^,^18^.

### Data availability

The code for our single-cell model of the electrical activity of SCN neurons with *I*_NaP_ has been deposited on ModelDB (accession number: 196197). From this code, the single cell and network behaviour can be generated. Details of our model network simulations can be found in ref. 36. The data that support the findings of this study are available from the corresponding authors upon reasonable request.

## Additional information

**How to cite this article:** Paul, J. R. *et al.* Regulation of persistent sodium currents by glycogen synthase kinase 3 encodes daily rhythms of neuronal excitability. *Nat. Commun.* **7,** 13470 doi: 10.1038/ncomms13470 (2016).

**Publisher's note:** Springer Nature remains neutral with regard to jurisdictional claims in published maps and institutional affiliations.

## Supplementary Material

Supplementary Information Supplementary Figures 1-6 and Supplementary Table 1
