# Supplementary material for: Regulation of persistent sodium currents by glycogen synthase kinase 3 encodes daily rhythms of neuronal excitability
Source: Nat Commun. 2016 Nov 14;7:13470. doi: 10.1038/ncomms13470 (PMC5114562; doi:10.1038/ncomms13470)
Supplement: Supplementary Information — Supplementary Figures 1-6 and Supplementary Table 1 [file ncomms13470-s1.pdf]

SUPPLEMENTARY INFORMATION

Supplementary Figures

Supplementary Figure 1

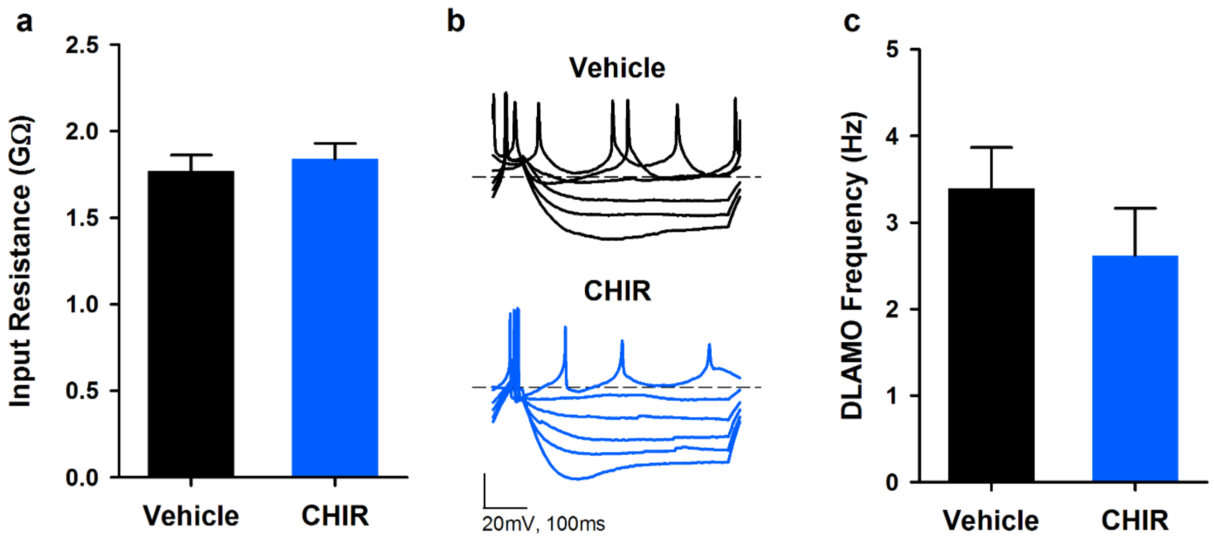

Supplementary Figure 1. GSK3 inhibition does not alter input resistance or DLAMO frequency.

(**a**) Bar graph (mean ± SEM) for input resistance of vehicle and CHIR treated SCN cells recorded during the day (independent samples t-test; $t_{(64)} = -0.562$, $p = 0.576$; n = 31-35 cells, 3-4 animals per group). (**b**) Representative current clamp traces from vehicle and CHIR treated SCN cells which were injected with progressive steps of hyperpolarizing current (5 pA steps from -25 pA to 0 pA). (**c**) Bar graph (mean ± SEM) for oscillation frequency of vehicle and CHIR treated SCN cells exhibiting DLAMO's (independent samples t-test; $t_{(27)} = 1.061$, $p = 0.298$; n = 13-16 cells, 3-4 animalsper group).

Supplementary Figure 2

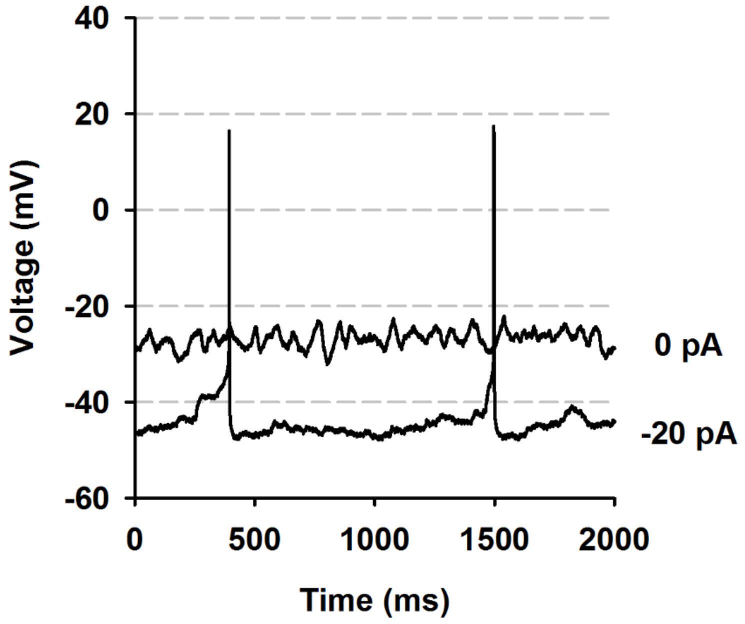

Supplementary Figure 2. Representative SCN neuron exhibiting depolarization block.

Representative current clamp trace from SCN neuron exhibiting depolarization block with no current injection (0 pA). Spontaneous AP firing was restored upon injection of 20 pA of negative current to hyperpolarize the neuron.

Supplementary Figure 3

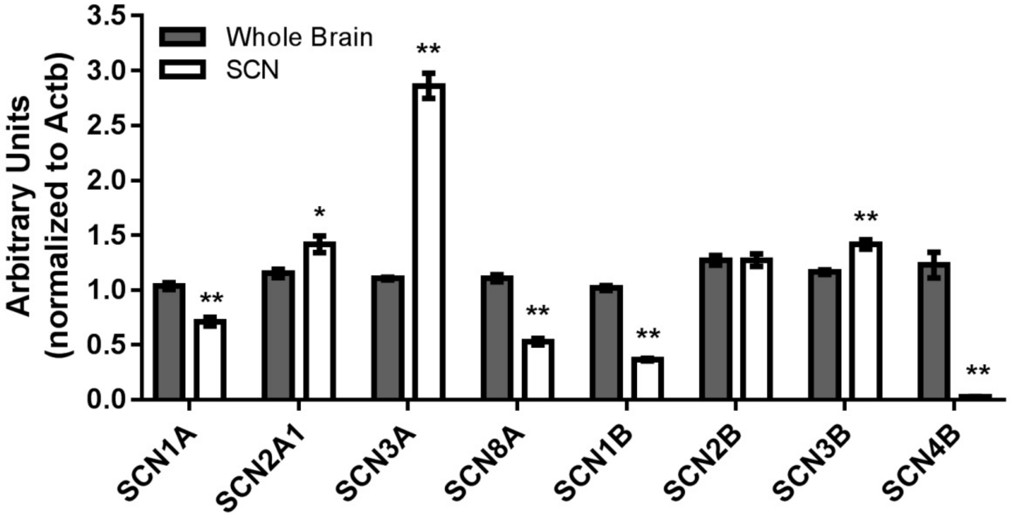

Supplementary Figure 3. RT-PCR results for voltage gated sodium channel α and β subunits in SCN.

To determine the enrichment of voltage-gated sodium channels in the SCN, transcript levels for alpha and beta subunits in isolated SCN were compared to that of the whole brain by q-RT-PCR. Transcript levels for Scn1a (Na_V1.1), Scn8a (Na_V1.6), Scn1b (β1) and Scn4b (β4) show significantly reduced expression levels in the SCN relative to whole brain, while that of Scn2b (β2) shows no difference. Gene expression for Scn2a1 (Na_V1.2), Scn3a (Na_V1.3), and Scn3b (β3) show significantly higher transcript levels in the SCN relative to whole brain RNA, indicating enrichment for these transcripts in SCN. n=6/group; 2 SCN pooled per sample. *p<0.05; **p<.01; two-tailed t test or Mann-Whitney U test. Data are presented as mean ± SEM.

Supplementary Figure 4

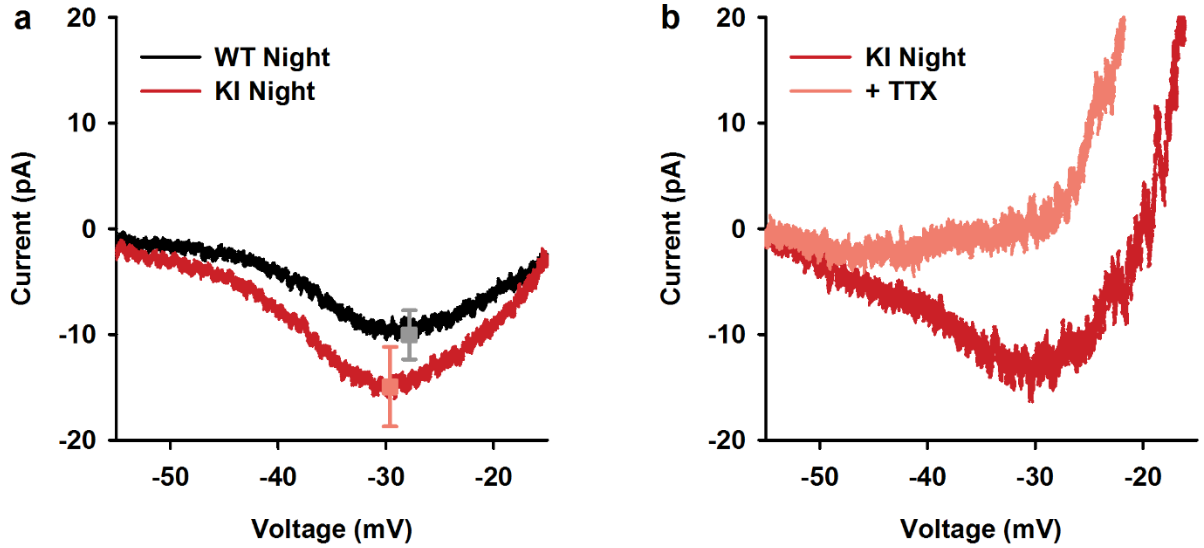

Supplementary Figure 4. Calcium and potassium channel blockers do not alter enhanced inward current in GSK3-KI mice.

(**a**) Average normalized response to slow depolarizing voltage ramp (-100 to +10 mV; 59mV/s) of SCN cells from WT or GSK3-KI during the early-night in the presence of TEA (10mM) and $CdCl_2$ (0.1mM). Peak I_{NaP} (means ± SEM shown as black and red symbols) in these conditions were similar to those measured in normal saline (see Figure 3C). n = 5-7 cells, 2 animals per group. (**b**) Representative response to slow depolarizing ramp in GSK3-KI SCN cell in normal saline and after treatment with TTX (3 nM; Tocris Bioscience, Bristol, UK).

Supplementary Figure 5

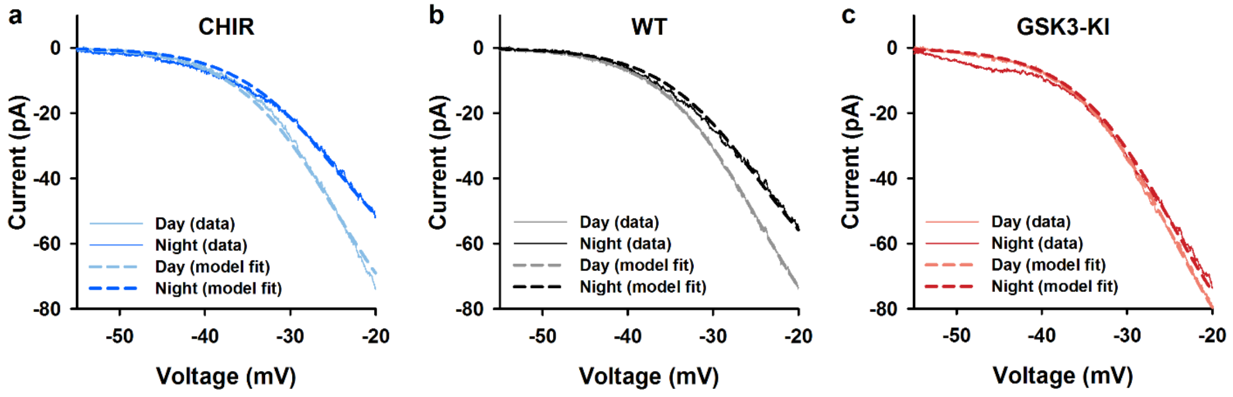

Supplementary Figure 5. Computational model fits experimental data of the circadian change in peak I_{NaP}.

Voltage ramps used to fit our model of I_{NaP}. Data (solid lines), and model predictions (dashed) are compared in the CHIR (**a**), WT (**b**) and GSK3-KI (**c**) conditions. Day and night curves were fit to determine the circadian change in peak current.

Supplementary Figure 6

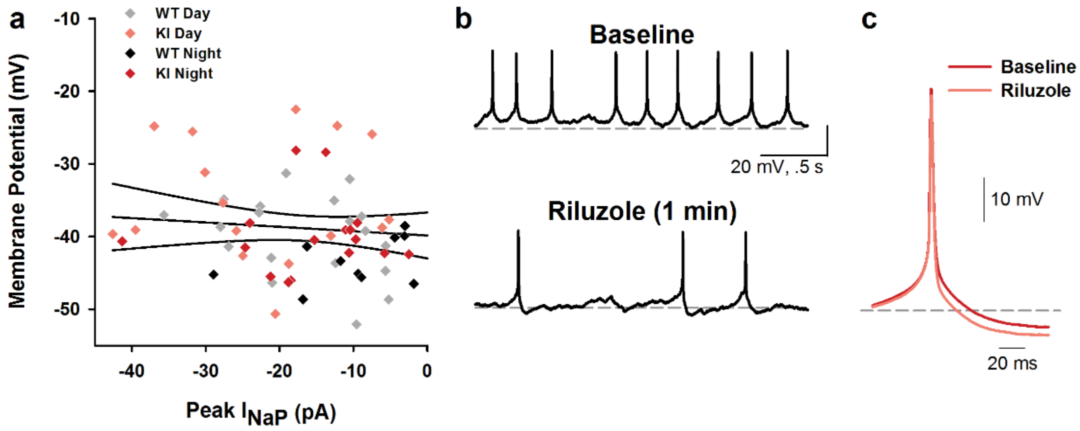

Supplementary Figure 6. RMP is not correlated with peak I_{NaP} in SCN neurons from WT and GSK3-KI mice.

(a) Pearson correlation, R = -0.094, p = 0.463. Lines represent linear fit and 95% confidence intervals for all cells in plot. WT day, n = 19 cells, 4 animals; WT night, n = 10 cells, 3 animals; GSK3-KI day, n = 18 cells, 3 animals; GSK3-KI night, n = 16 cells, 3 animals. (**b-c**) Representative current clamp traces (**b**) and action potential waveforms (**c**) from a GSK3-KI nighttime neuron before and after treatment with riluzole (10 µM). On average, riluzole treatment significantly increased the AHP amplitude (n = 6; paired samples t-test, $t_{(5)}$ = 6.470, p = 0.001), but had no effect on RMP ($t_{(5)}$ = -.552, p = .605).

Supplementary Tables

Supplementary Table 1. Primer/probes for Taqman

**Gene**	**Primer/probe**
β-actin	Mm00607939_s1 (Applied Biosystems)
Scn1a	Mm00450580_m1 (Applied Biosystems)
Scn1b	Mm00441210_m1 (Applied Biosystems)
Scn2a1	Mm01270359_m1 (Applied Biosystems)
Scn2b	Mm01179204_g1 (Applied Biosystems)
Scn3a	Mm00658167_m1 (Applied Biosystems)
Scn3b	Mm00463369_m1 (Applied Biosystems)
Scn4b	Mm01175562_m1 (Applied Biosystems)
Scn8a	Mm00488110_m1 (Applied Biosystems)
